# A novel peptide Phylloseptin‐PBu from *Phyllomedusa burmeisteri* possesses insulinotropic activity via potassium channel and GLP‐1 receptor signalling

**DOI:** 10.1111/jcmm.13573

**Published:** 2018-03-07

**Authors:** Qilin Long, Lei Wang, Mei Zhou, Yuxin Wu, Tianbao Chen

**Affiliations:** ^1^ Natural Drug Discovery Group School of Pharmacy Queen's University Belfast Belfast UK

**Keywords:** calcium, GLP‐1 receptor, insulin release, peptide, PKA signalling, potassium channel

## Abstract

Insulin, as one of the most important hormones regulating energy metabolism, plays an essential role in maintaining glucose and lipid homeostasis *in vivo*. Failure or insufficiency of insulin secretion from pancreatic beta‐cells increases glucose and free fatty acid level in circulation and subsequently contributes to the emergence of hyperglycaemia and dyslipidaemia. Therefore, stimulating the insulin release benefits the treatment of type 2 diabetes and obesity significantly. Frog skin peptides have been extensively studied for their biological functions, among which, Phylloseptin peptides discovered in Phyllomedusinae frogs have been found to exert antimicrobial, antiproliferative and insulinotropic activities, while the mechanism associated with Phylloseptin‐induced insulin secretion remains elusive. In this study, we reported a novel peptide named Phylloseptin‐PBu, isolated and identified from *Phyllomedusa burmeisteri*, exhibited dose‐dependent insulinotropic property in rat pancreatic beta BRIN‐BD11 cells without altering cell membrane integrity. Further mechanism investigations revealed that Phylloseptin‐PBu‐induced insulin output is predominantly modulated by K_ATP_‐[K^+^] channel depolarization triggered extracellular calcium influx and GLP‐1 receptor initiated PKA signalling activation. Overall, our study highlighted that this novel Phylloseptin‐PBu peptide has clear potential to be developed as a potent antidiabetic agent with established function‐traced mechanism and low risk of cytotoxicity.

## 1 INTRODUCTION

Type 2 diabetes mellitus (T2DM), accounting for almost 90% cases of the disease, has become a global public health threat for both individuals and social healthcare systems.^1^ Although the aetiology of T2DM has not been fully understood, it is widely accepted that deficiency of insulin secretion from pancreatic beta‐cells accompanied by attenuation/abnormality of insulin sensitivity in specific tissues (liver, heart, fat, skeleton muscle) is the common feature of T2DM.^2^ Either beta‐cell disorder or decreased beta‐cell mass contributes to the impairment of insulin release.^3^ Hyperglycaemia and dyslipidaemia, as hallmarks of T2DM, appear when the blood glucose and lipid lowering effect of secreted insulin is not sufficient to compensate for insulin resistance. When patients suffer the long‐term pathological conditions, complications of T2DM, such as heart and blood vessel diseases, neuropathy, nephropathy, eye/foot/hearing/skin damage and Alzheimer's disease, will occur.^4^, ^5^, ^6^

Clinically, the therapies for the treatment of T2DM are aimed at decreasing hepatic glucose production, improving insulin resistance and increasing insulin release. In particular, for the insulin secretion boosting therapy, two types of drugs are currently prescribed, one acts on closing the K_ATP_ channels on the beta‐cells for the calcium influx, and the other type targets on mimicking or enhancing the effect of gut hormones (also named as incretins),^7^ a good example of the latter type is extendin‐4, it was firstly isolated and characterized from the *Heloderma suspectum* venom, the glycaemic‐control action of extendin‐4 is via the similar way with glucagon‐like peptide 1 (GLP‐1), which mainly attributes to the potentiation of insulin secretion in a glucose‐sensing manner.^7^, ^8^, ^9^, ^10^, ^11^, ^12^, ^13^, ^14^

Bioactive peptides from amphibian secretions and venoms with characteristics of diversity and specificity provide candidates for the development of novel drugs for combatting multiple diseases, including antibiotic‐resistant pathogens caused infections, cancers and T2DM.^15^ Previous elegant research has reported that some frog skin‐derived peptides possess the ability to stimulate insulin release both *in vitro* and *in vivo* at relatively low concentration and dose without causing cellular toxicity.^16^, ^17^, ^18^ Many have been extensively studied in the fields of amino acid isoform replacement, post‐translational modification and primary structure modification, all of these focus on increasing the insulin release efficiency, preventing the degradations caused by endogenous enzymes and improving the cytotoxicity.^16^, ^19^, ^20^ Nevertheless, the mechanism associated with how these bioactive peptides are involved in the insulin release action has rarely been investigated. Thus, in this study, for the first time, we reported a novel insulinotropic peptide isolated from *Phyllomedusa burmeisteri*, in which species such peptide has never been identified, belongs to Phylloseptin family, named as Phylloseptin‐PBu. At low concentration, it showed glucose‐comparably insulin secretion stimulatory effects in pancreatic beta BRIN‐BD11 cells. Cell signalling examinations revealed the involvements of K_ATP_‐[K^+^] channel‐induced intracellular calcium elevation and partial GLP‐1 receptor activation‐induced PKA stimulation, which is firstly reported. Therefore, we not only provide a novel candidate for developing antidiabetic agent, but also elucidate the preliminary mechanism of its bioactivity, illuminating the future work for promoting this agent into clinical tests.

## 2 MATERIALS AND METHODS

### 2.1 Acquisition of frog skin secretions

The specimens of Burmeister's leaf frog, *Phyllomedusa burmeisteri* (n = 3, 6 and 8 cm snout‐to‐vent lengths), were sampled in the Atlantic Forest biome of Brazil. The skin secretions were obtained by mild transdermal electrical stimulation (5 V, 50 Hz, 4 ms pulse width).^21^ The skin secretions were then collected by rinsing with distilled deionized water and subjected to snap frozen with liquid nitrogen, lyophilized and stored at −20°C prior to use.

### 2.2 “Shotgun” cloning of a novel Phylloseptin‐like peptide from skin secretion‐derived cDNA library

Five milligrams of lyophilized *Phyllomedusa burmeisteri* secretion powder was dissolved in 1 mL cell lysis/binding buffer to isolate polyadenylated mRNA by magnetic oligo‐dT beads via using Dynabeads^®^ mRNA DIRECT™ Kit (Dynal Biotech, Liverpool, UK). The reverse‐transcription products were subjected to 3′‐RACE PCR procedures to acquire the full length of preproprotein nucleic acid sequences using a SMART‐RACE kit (Clontech, Palo alto, CA, USA). Briefly, a nested universal primer (NUP) (supplied with the kit) and a degenerate primer (S1; 5′‐ACTTTCYGAWTTRYAAGMCCAAABATG‐3′, Y = C + T, W = A + T, R = A + G, M = A + C, B = T + C + G) which was designed on the basis of the 5′‐untranslated region of phylloxin cDNA from *Phyllomedusa bicolor*.^22^ The PCR cycling was carried out as follows: initial denaturation step: 90 seconds at 94°C; 35 cycles: denaturation 30 seconds at 94°C, primer annealing for 30 seconds at 58°C; extension for 180 seconds at 72°C. PCR products were gel‐purified and cloned using a pGEM‐T Easy vector system (Promega Corporation, Southampton, UK), and then, selected samples were sequenced using an ABI 3100 automated sequencer (Applied Biosystems, Foster City, CA, USA).

### 2.3 Identification and structural characterization of the novel Phylloseptin‐like peptide from *Phyllomedusa burmeisteri* skin secretion

An aliquot sample of lyophilized skin secretion was dissolved in 1 mL trifluoroacetic acid (TFA)/water (0.05/99.95, v/v) and then clarified by centrifuge. One millilitre supernatant was decanted and pumped into reversed phase‐HPLC column (Phenomenex C‐5, 300 Å, 5 μm, 4.5 × 250 mm, Phenomenex, Macclesfield, Cheshire, UK) using a gradient program. The gradient elution formed from TFA/water (0.05/99.95, v/v) to TFA/water/Acetonitrile (0.05/19.95/80.00, v/v/v) in 240 minutes at a flow rate of 1 mL/min, and the effluent was detected by UV absorbance at 214 and 280 nm. Fractions were collected at 1‐minutes interval by an automatic fraction collector (GE Healthcare, Little Chalfont, UK). The molecular mass of each fraction was analysed using the matrix‐assisted laser desorption ionization time of flying (MALDI‐TOF) mass spectrometer (Voyager DE, PerSeptive Biosystems, Foster City, CA, USA) with α‐cyano‐4‐hydroxycinnamic acid (CHCA) matrix in positive detection mode. Fractions with the molecular weight coincidence with the mature peptide which is predicted from cloned cDNA were then subjected to primary structural analysis by MS/MS fragmentation using a liquid chromatography quadrupole (LCQ)‐Fleet electrospray ion‐trap mass spectrometer (Thermo Fisher Scientific, San Francisco, CA, USA).

### 2.4 Solid phase peptide synthesis of Phylloseptin‐PBu

Once the primary structural of the Phylloseptin‐PBu was unequivocally confirmed through both MS/MS fragmentation sequencing and molecular cloning, the solid‐phase Fmoc strategy was adopted via Tribute™ automated solid‐phase peptide synthesizer 4 (Protein Technologies, Tucson, AZ, USA) to synthesize the novel peptide as previously described.^23^ The synthesized peptide replicates were purified by reverse phase HPLC with C18 column (Jupiter C18, 5 μm particle, 300 A pore, 250 × 10 mm, Phenomenex, UK), and both molecular masses and high‐purity of the synthetic replicates were confirmed via MALDI‐TOF.

### 2.5 Determination of peptide secondary structures using circular dichroism (CD) analysis

CD measurements were performed using a JASCO J‐815 CD spectrometer (Jasco, Essex, UK) at room temperature (20‐25°C). Peptide was dissolved in (*i*) water, (*ii*) 50% (v/v) trifluoroethanol (TFE)‐water and (*iii*) 50% (v/v) Methanol‐water to reach a final concentration of 100 μmol/L before transferred and measured in a 0.1‐cm‐high precision quartz cell (Hellma Analytics, Essex, UK). The wavelength was from 190 to 260 nm with a scanning speed of 200 nm/min, the bandwidth and data pitch are 1 and 0.5 nm, respectively. CD data are expressed as the mean residue ellipticity [θ] in deg.cm^2^.dmol^−1^ in corresponding wavelength (nm), which is calculated from the measured ellipticity (θ, in medg) using the equation [θ] = θ/(10 × c × l), where “c” is the molar concentration of the sample (M) and “l” is the cuvette path length in centimetre. DICHROWEB webserver (http://dichroweb.cryst.bbk.ac.uk/html/home.shtml) was used to estimate the α‐helix and β‐sheet content.^24^, ^25^, ^26^

### 2.6 Cell line and cell culture

The BRIN‐BD11 rat clonal beta‐cell line is purchased from European Collection of Authenticated Cell Cultures (Catalogue No. 10033003, UK), which serves as a canonical in vitro model to study insulin release in response to nutrients and chemicals.^27^ After resuscitation, BRIN‐BD11 cells were cultured using RPMI‐1640 medium (Invitrogen, Paisley, UK) containing 10% (v/v) foetal bovine serum (FBS) (gibco^®^, UK), antibiotics (100 U/mL penicillin, 0.1 mg/mL streptomycin, gibco^®^, UK) and 11.1 mmol/L D‐glucose at 37°C with 5% CO_2_.

### 2.7 Measurement of acute insulin secretion using rat insulin ELISA

BRIN‐BD11 cells were seeded into 24‐well plates (Nunc, Roskilde, Denmark) with the density of 3 × 10^5^ cells/well for 24‐hours incubation. After then, cells were pre‐incubated for 40 minutes at 37°C in Krebs Ringer Bicarbonate (KRB) buffer (115 mmol/L NaCl, 4.7 mmol/L KCl, 1.28 mmol/L CaCl_2_, 1.2 mmol/L KH_2_PO_4_, 1.2 mmol/L MgSO_4_,10 mmol/L NaHCO_3_, pH 7.4) supplemented with 1.1 mmol/L glucose and 0.1% (w/v) bovine serum albumin (BSA) (Sigma, UK). Acute administration of a concentration gradient (10^−12^ to 10^−7^ mol/L) of purified synthetic Phylloseptin‐PBu was performed (n = 5) in the presence of 5.6 mmol/L or 16.7 mmol/L glucose in KRB buffer for 20 minutes at 37°C. After which, aliquots of cell culture supernatant from each well were collected and measured using Ultra‐Sensitive Rat Insulin ELISA kit (Crystal Chem Inc, USA) according to the manufacturer's instruction.

For cell signalling studies, 300 μmol/L diazoxide (Sigma, UK), 60 μmol/L verapamil (Sigma, UK), 200 μmol/L IBMX (Tocris Bioscience, UK), 10 μmol/L H89 (Tocris Bioscience, UK) and 1 μmol/L Exendin (9‐39) amide (Tocris Bioscience, UK) were pre‐treated 30 minutes before Phylloseptin‐PBu stimulation unless specific states.

All cell culture designs for the ELISA measurement were repeated with three independent studies, and every study contains three replicates for each treatment.

### 2.8 Cell membrane integrity, haemolytic and antimicrobial evaluations

The lactate dehydrogenase (LDH) assay was employed to assess the cell membrane integrity after Phylloseptin‐PBu treatment. The rate of LDH release from BRIN‐BD11 cells was measured using Pierce LDH Cytotoxicity Assay Kit (Thermo scientific, USA) according to the manufacturer's instruction with the cell culture supernatant collected from acute insulin release assay.

Peptide concentrations range from 10^−7^ to 10^−12^ mol/L was prepared for assessing the haemolytic and antimicrobial properties of Phylloseptin‐Pbu, and the detailed procedures were described previously.^28^

### 2.9 Western blotting

BRIN‐BD11 cells were seeded into 100‐mm tissue culture dishes at a density of 2 × 10^6^ cells/dish for 24 hours prior to 6‐hours serum‐free starvation, Phylloseptin‐PBu (10^−7^ mol/L) was then added for 16‐hours incubation. The cells were rinsed twice with ice‐cold PBS, solubilized in lysis buffer containing 50 mmol/L Tris (pH 7.5), 1% Nonidet P‐40, 150 mmol/L NaCl, 2 mmol/L EGTA, 1 mmol/L Na_3_VO_4_, 100 mmol/L NaF, 10 mM Na_4_P_2_O_7_, 1 mmol/L phenylmethylsulfonyl fluoride, 10 μg/mL aprotinin and 10 μg/mL leupeptin. After centrifugation at 14 000× g for 10 minutes at 4°C, the supernatant was boiled for 5 minutes in SDS‐PAGE sample buffer [50 mmol/L Tris‐Cl (pH 6.8), 2% sodium dodecyl sulphate, 10% glycerol, 2% β‐mercaptoethanol and 0.004% bromophenol blue] and separated by 7.5% SDS‐PAGE, and transferred to nitrocellulose membrane (GE Healthcare Life sciences, UK), and detected by Western blotting with the indicated antibody (Cell Signaling Technology, UK) using enhanced chemiluminescence [Pierce^®^ ECL Western Blotting Substrate (Thermo scientific, USA)], and imaged via Chemi Doc™ MP system (Bio‐Rad, UK).

The antibodies that chosen for the present study were PKA‐Cα (1:1000; Cell Signaling, #4782), phospho‐PKA‐C (Thr197) (1:1000; Cell Signaling, #5661), GAPDH (1:1000; Cell Signaling, #5174) and Anti‐rabbit IgG, HRP‐linked Antibody (1:2000; Cell Signaling, #7074).

### 2.10 Statistical analysis

All results are presented as mean ± SEM determined by two‐tailed Student's *t* test or one‐way ANOVA. Pairs comparisons of the means were made, and *P* < .05 was regarded as a significant difference. The Bonferroni method was used to adjust the observed significance levels for the fact the multiple contrasts were being tested.

## 3 RESULTS

### 3.1 Primary structure identification and synthesis of novel peptide Phylloseptin‐PBu

**Figure 1:**
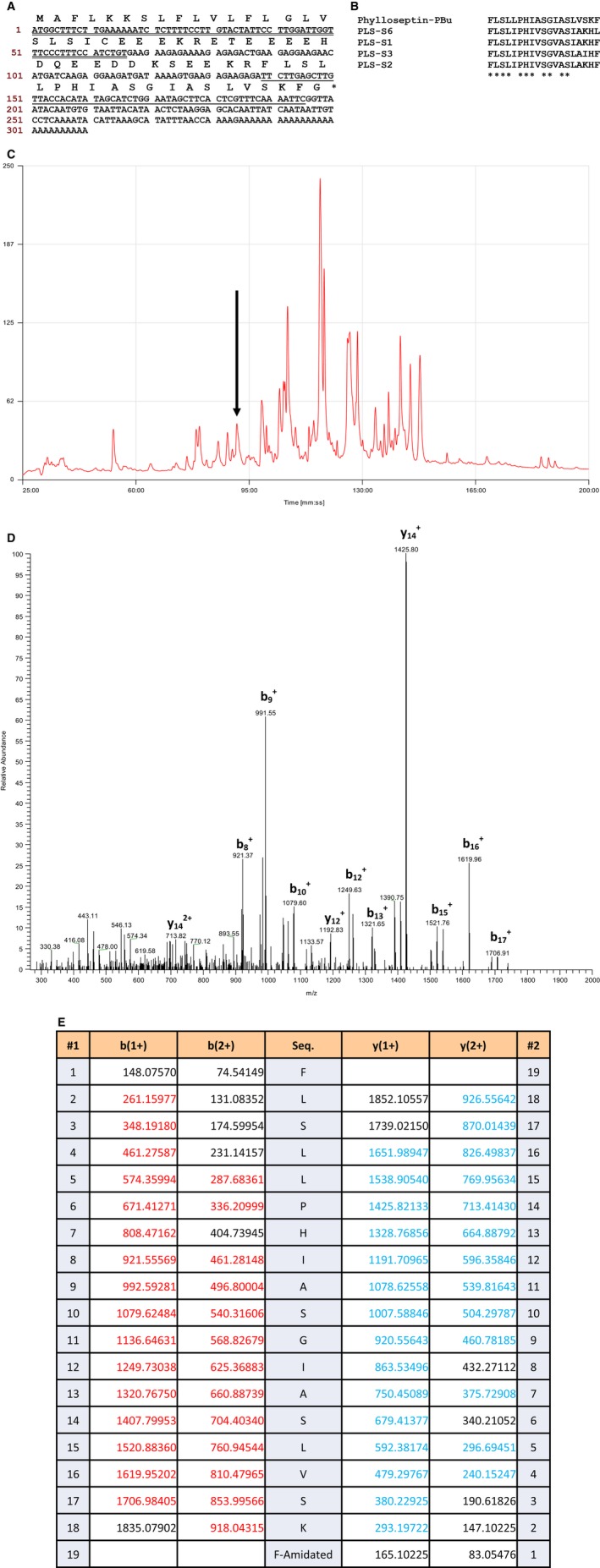
A, Nucleotide and translated open‐reading frame amino acid sequences of full‐length prepro‐Phylloseptin‐PBu peptide encoding cDNA from *Phyllomedusa burmeisteri* (EMBL accession number: LT841135). The putative signal peptide is double‐underlined, the mature peptide is single‐underlined and the stop codon is indicated by an asterisk. B, Alignment of cDNA deduced mature Phylloseptin‐PBu sequence with phylloseptin peptides from *Phyllomedusa sauvagii* species. Highly conserved residues are indicated by asterisks (*). C, Region of rp‐HPLC chromatogram of *Phyllomedusa burmeisteri* skin secretion with arrow indicating the retention times (at 91 min) of the novel peptide Phylloseptin‐PBu. The detection wavelength was 214 nm with a flow rate of 1 mL/min in 240 min. D, Thermoquest LCQ™ fragment scan spectrum derived from ions corresponding to Phylloseptin‐PBu. E, Expected single‐ and double‐charged b‐ and y‐ion series predicted from LCQ MS/MS fragmentation of Phylloseptin‐PBu. The fragment ions observed following actual fragmentation are shown in red (b‐ions) and blue (y‐ions) typefaces

From the skin secretion of *Phyllomedusa burmeisteri*‐derived cDNA library, the cDNA encoding the biosynthetic precursor of Phylloseptin‐PBu consistently and repeatedly cloned. The coding region of the cDNA consisted of 66 amino acids which can be divided into four domains: the putative signal peptide, which is located at the 5′‐termius with 22 amino acids; the 24‐residue acidic spacer domain, a typical Lys‐Arg (K‐R) propeptide convertase processing site and the 19‐mer mature peptide named Phylloseptin‐PBu (FLSLLPHIASGIASLVSKF‐NH_2_) (Figure 1A). The peptide Phylloseptin‐PBu sequence showed high similarity with phylloseptin‐S6 (EMBL accession number: HE974361), phylloseptin‐S1 (AM903077), phylloseptin‐S3 (AM903079) and phylloseptin‐S2 (AM903078) from *Phyllomedusa sauvagii* (Figure 1B). The nucleotide sequence of the cDNA encoding the novel Phylloseptin‐PBu precursor from the skin secretion of *Phyllomedusa burmeisteri* has been deposited in the EMBL Nucleotide Sequence Database under the accession code: LT841135.

The rp‐HPLC fractions collected from crude skin secretion of *Phyllomedusa burmeisteri* (Figure 1C) were all analysed by matrix assisted laser desorption ionization time of flight (MALDI‐TOF) mass spectrometry to determine the molecular mass. The effluent showed the same mass with the putative peptide from molecular cloning was subjected to liquid chromatography quadrupole (LCQ)‐Fleet electrospray ion‐trap mass spectrometer for primary structural determination (Figure 1D,E). The mature peptide sequence of Phylloseptin‐PBu ended with amidation, a post‐translational modification at C‐terminus, was successfully detected by LCQ MS/MS. Taken together, the primary sequence of was unequivocally determined as FLSLLPHIASGIASLVSKF‐NH_2_.

Phylloseptin‐PBu peptide replicates were then synthesized by standard solid‐phase Fmoc chemistry using Tribute™ automated solid‐phase peptide synthesizer 4 (Protein Technologies, Tucson, AZ, USA). Following cleavage from the synthesis resin, impurities were removed from the synthetic replicates by rp‐HPLC and the molecular masses of the purified major products were confirmed by MALDI‐TOF mass spectrometry (Figure S1).

### 3.2 Secondary structure determination by circular dichroism analysis

**Figure 2:**
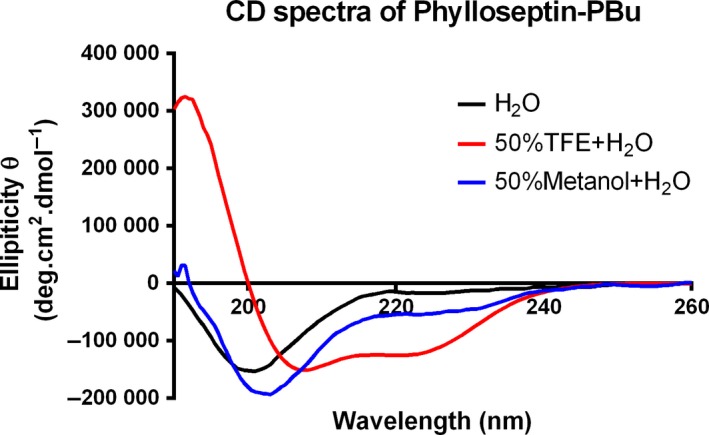
The Circular Dichroism spectra of Phylloseptin‐PBu in H_2_O, 50% 2,2,2‐trifluoroethanol (TFE)‐H_2_O and 50% Methanol‐H_2_O

The CD spectra (Figure 2) indicated that Phylloseptin‐PBu displayed diverse secondary structure conformations in different surroundings. In aqueous solution, a mixed conformation of random coil (42%) and β‐sheet (51%) was presented with a negative band at 200 nm. A similar conformation was observed in 50% methanol‐water solution with a slightly higher presence of random coil. By contrast, a typical α‐helical conformation was shown in 50% TFE‐water solution with one positive band at 192 nm and two negative bands at 206 nm and 222 nm, respectively, and the predicted α‐helical contents of Phylloseptin‐PBu were 96% in 50%TFE‐water solution. The percentage of secondary structure helicity of Phylloseptin‐PBu in respective solutions are shown in Table 1.

**Table 1:** Helicity index of Phylloseptin‐PBu analysed according to CD spectra data using DICHROWEB online server

Percentage of helicity (%)	α‐helix	β‐sheet	Random coil
H_2_O	7	51	42
50% TFE + H_2_O	96	0	4
50% Methanol + H_2_O	8	45	47

### 3.3 Phylloseptin‐PBu induces insulin release from BRIN‐BD11 cells

**Figure 3:**
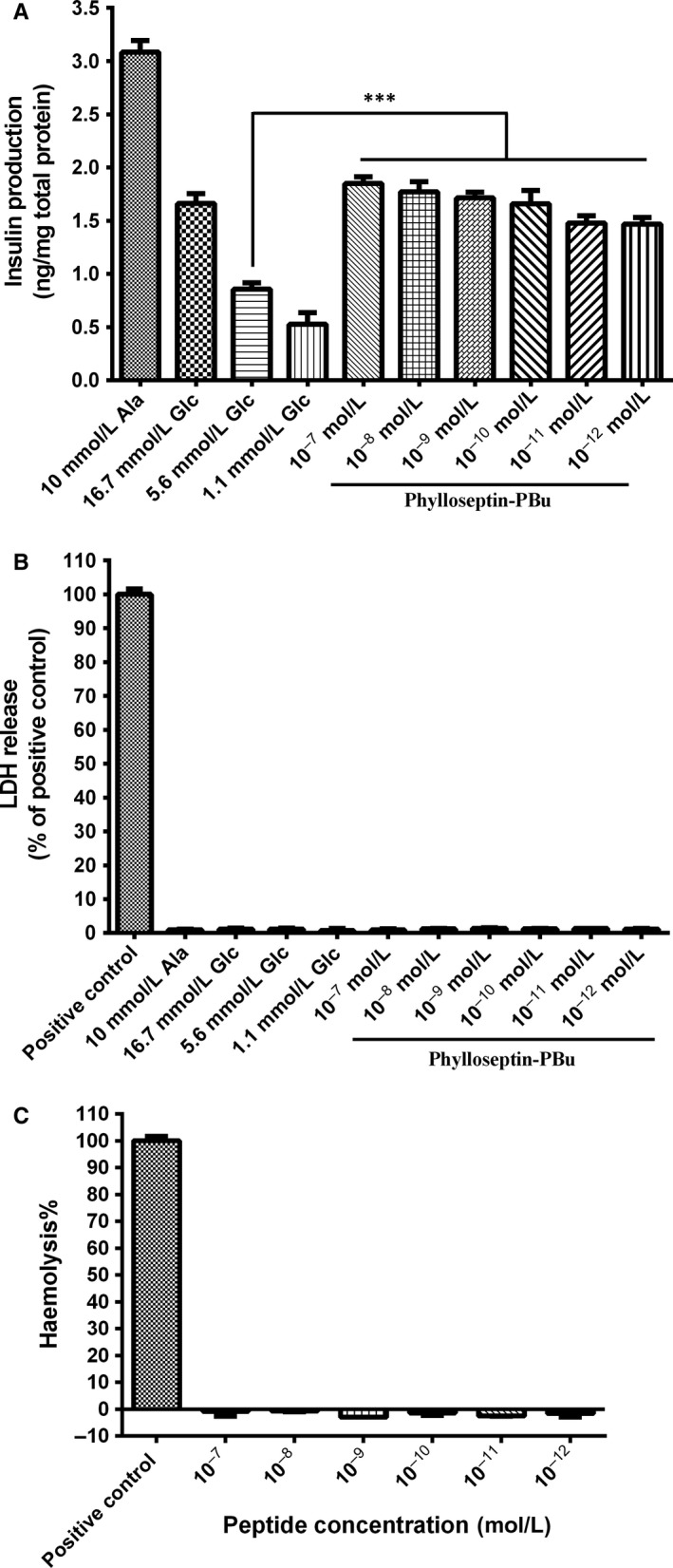
A, Acute effects (20 min stimulation) of a range concentration (10^−12^ to 10^−7^ mol/L) of Phylloseptin‐PBu peptide on insulin secretion from BRIN‐BD11 cells. ***P < .001 vs 5.6 mmol/L glucose alone. B, Lactate dehydrogenase (LDH) release from BRIN‐BD11 cells after acute stimulation (20 min) of 10 mmol/L alanine and a range concentration (10^−12^ to 10^−7^ mol/L) of Phylloseptin‐PBu. C, the haemolytic activity of a range concentration (10^−12^ to 10^−7^ mol/L) of Phylloseptin‐PBu was tested on horse red blood cells. The positive control group was treated with 1% Triton X‐100 lysis buffer. Ala, Alanine; Glc, Glucose

To investigate whether Phylloseptin‐PBu could stimulate insulin release, we treated rat pancreatic beta BRIN‐BD11 cells with a concentration range from 10^−7^ to 10^−12^ mol/L, along with a 5.6 mmol/L glucose group, which mimics the glucose level in physiological conditions, a 16.7 mmol/L glucose group, which is a stimulatory glucose level (suggesting diabetic ketoacidosis), and a positive alanine group. The results (Figure 3A) showed that, upon Phylloseptin‐PBu stimulation, BRIN‐BD11 cells exhibited a significant higher, and a dose‐dependent, insulin release capacity compared with 5.6 mmol/L glucose group, this effect was even stronger than 16.7 mmol/L glucose group when administered the 10^−7^ or 10^−8^ mol/L concentration of the peptide. Although the insulinotropic effect of Phylloseptin‐PBu is weaker compared to L‐alanine, which mainly induces insulin output via Ca^2+^ influx mechanism,^29^ it still prompted us to explore the signal pathways that involved in this novel Phylloseptin‐PBu peptide exhibited bioactivity.

As Phylloseptin‐like peptides have been reported to exert antimicrobial activities against Gram‐positive and Gram‐negative bacterial and fungi by disrupting the cell membrane.^30^ We therefore investigated whether Phylloseptin‐PBu‐triggered insulin release was caused by the damage to cell membrane. The minimal inhibitory concentration (MIC) assay implied that Phylloseptin‐PBu possessed no antimicrobial activity against *S. aureus*,* E. coli* and *C. albicans* when the concentration was up to 10^−7^ mol/L (Figure S2). The lactate dehydrogenase (LDH) tests suggested that Phylloseptin‐PBu treatment did not compromise cell membrane integrity in BRIN‐BD11 cells at concentrations up to 10^−7^ mol/L (Figure 3B). Further haemolytic measurement using horse blood cell also did not show cytolytic effect of Phylloseptin‐PBu at 10^−7^ mol/L (Figure 3C). Therefore, these results lead us to investigate the specific mechanism that related to insulin release effect of Phylloseptin‐PBu.

### 3.4 Potassium and calcium channel‐modulated Ca^2+^ influx examination

**Figure 4:**
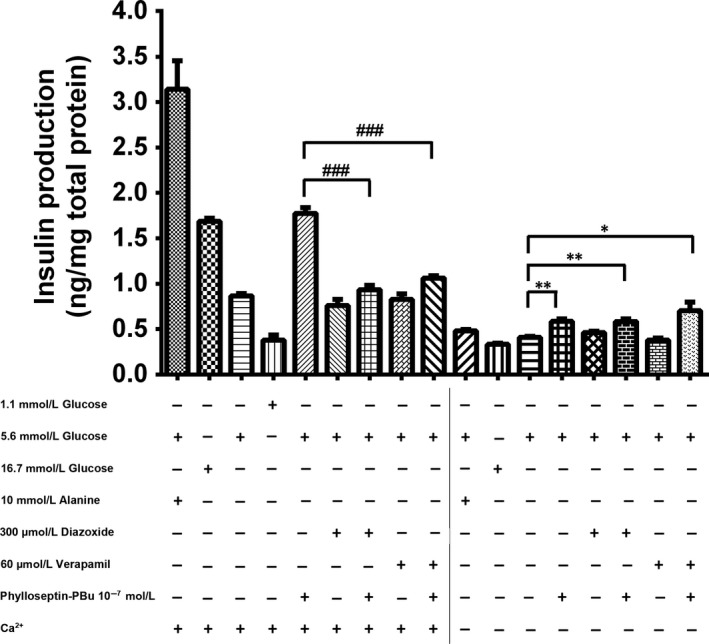
Acute effects (20 min stimulation) of 10^−7^ mol/L Phylloseptin‐PBu on the insulin release. BRIN‐BD11 cells were incubated with 1.1 mmol/L glucose, 5.6 mmol/L glucose, 16.7 mmol/L glucose, 10 mmol/L alanine, 300 μmol/L diazoxide, 60 μmol/L verapamil and 10^−7^ mol/L Phylloseptin‐PBu in the presence or absence of Ca^2+^ KRB buffer. Data were analysed with unpaired Student's *t* test using GraphPad Prism 5 software. Values are the mean ± SEM for three independent experiments. *P < .05, ** or ^##^ *P* < .01, ^###^ *P* < .001 between indicated groups

As indicated, elevated intracellular ATP level and increased ATP/ADP ratio leads to the closure of potassium channel, induction of cell membrane depolarization and opening of voltage‐gated calcium channel. These actions promote extracellular Ca^2+^ influx, which plays a key role in mediating L‐alanine‐activated insulin release from BRIN‐BD11 cells.^31^ We employed diazoxide (activator of ATP‐sensitive potassium channels), verapamil (inhibitor of voltage‐gated calcium channels) and Ca^2+^‐free medium to examine whether Phylloseptin‐PBu stimulated insulin release is via Ca^2+^ mobilization. As shown in Figure 4, pre‐administration of diazoxide and verapamil significantly attenuated Phylloseptin‐PBu‐induced insulin production, deprivation of Ca^2+^ significantly attenuated the insulin producing activity in all tested groups, but significant enhancement of insulin release was still observed after Phylloseptin‐PBu treatment even culturing the cells with Ca^2+^‐free KRB buffer with or without diazoxide or verapamil. Together, these results suggested that calcium influx, mediated by ATP‐sensitive potassium channel induced depolarization and calcium channel opening, played the dominant role in the insulinotropic activity of Phylloseptin‐PBu, nevertheless, this effect was not completely abrogated, it prompted us to hypothesize that other signalling might be involved.

### 3.5 PKA signalling activation‐modulated insulin secretion examination

**Figure 5:**
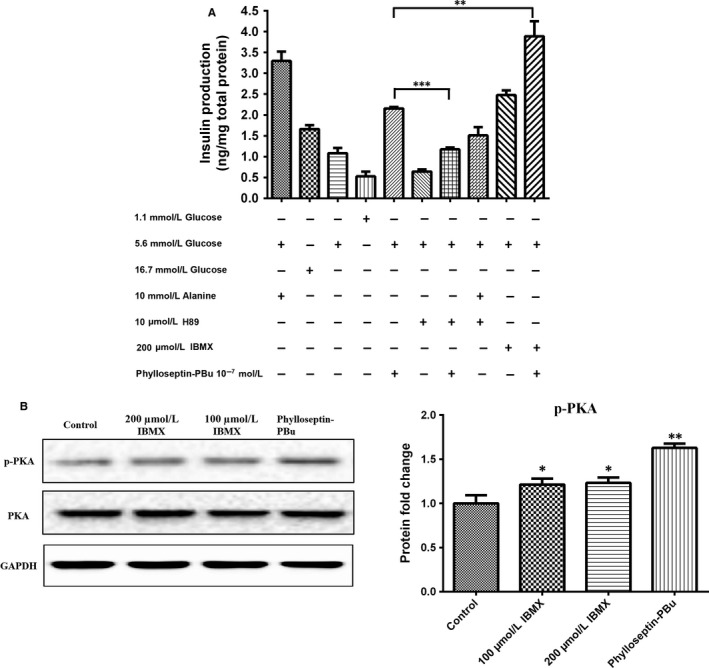
A, Acute effects (20 min stimulation) of 10^−7^ mol/L Phylloseptin‐PBu on the insulin release. BRIN‐BD11 cells were incubated with 1.1 mmol/L glucose, 5.6 mmol/L glucose, 16.7 mmol/L glucose, 10 mmol/L alanine, 10 μmol/L H89, 200 μmol/L IBMX and 10^−7^ mol/L Phylloseptin‐PBu in Ca^2+^‐contained KRB buffer. Data were analysed with unpaired Student's *t* test using GraphPad Prism 5 software. Values are the mean ± SEM for three independent experiments. **P < .01, ***P < .001 between indicated groups. B, BRIN‐BD11 cells were treated with 100, 200 μmol/L IBMX or 10^−7^ mol/L Phylloseptin‐PBu for 2 h, total proteins were extracted. Eighty μg protein was immunoblotted to determine the abundance of total PKA and phosphor‐PKA at Thr 197. Signal intensity was quantified by Image Lab software for statistical comparison, *P < .05, **P < .01 vs vehicle control

Activation of cAMP/PKA was also reported to elicit insulin secretion in islet.^32^ Thus, we interrogated the PKA signalling using the PKA activator (IBMX, 3‐Isobutyl‐1‐methylxanthine) and inhibitor (H89) in the presence or absence of Phylloseptin‐PBu. The results (Figure 5A) showed that although H89 significantly blunted Phylloseptin‐PBu‐induced insulin secretion, compared with singular H89 treatment, Phylloseptin‐PBu still has a trend to increase insulin release. While IBMX alone and co‐administered with Phylloseptin‐PBu increased and dramatically further enhanced insulin output, respectively. Based on these observations, we speculated that PKA signalling is not only modulated by Phylloseptin‐PBu, but also L‐alanine for the stimulation of insulin release from BRIN‐BD11 cells.

Furthermore, we used Western blotting to examine the effect of Phylloseptin‐PBu vs IBMX on PKA activation in BRIN‐BD11 cells. As shown in Figure 5B, the phosphorylation level of PKA at Thr197 was markedly elevated by IBMX and Phylloseptin‐PBu, which confirmed the involvement of PKA cascade in Phylloseptin‐PBu‐induced insulinotropic activity.

### 3.6 Assessment of GLP‐1 receptor activation by Phylloseptin‐PBu

**Figure 6:**
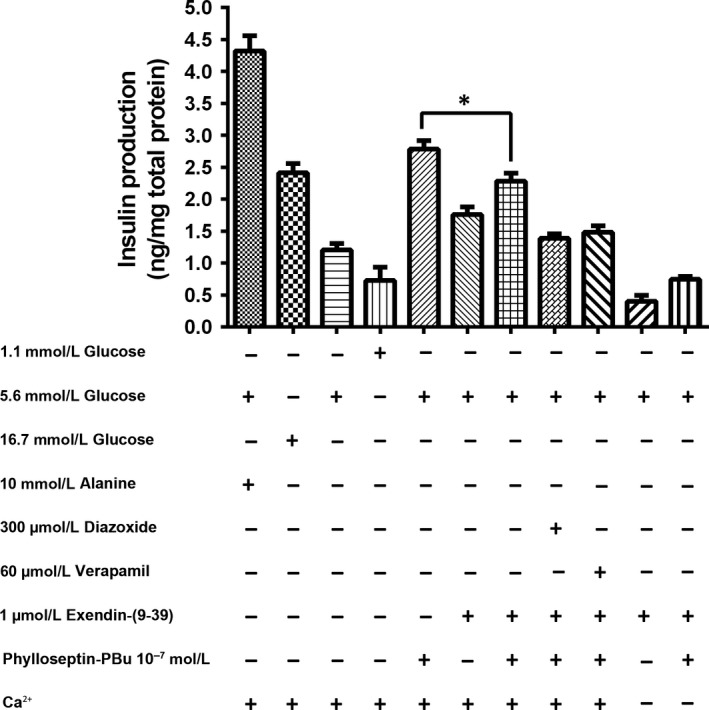
Acute effects (20 min stimulation) of 10^−7^ mol/L Phylloseptin‐PBu on the insulin release. BRIN‐BD11 cells were incubated with 1.1 mmol/L glucose, 5.6 mmol/L glucose, 16.7 mmol/L glucose, 10 mmol/L alanine, 300 μmol/L diazoxide, 60 μmol/L verapamil, 1 μmol/L exendin‐(9‐39) and 10^−7^ mol/L Phylloseptin‐PBu in the Ca^2+^ present or absent KRB buffer. Data were analysed with unpaired Student's *t* test using GraphPad Prism 5 software. Values are the mean ± SEM for three independent experiments. *P < .05 between indicated groups

Next, we investigated how Phylloseptin‐PBu activated PKA signalling. As GLP‐1 and extendin‐4 have been demonstrated to enhance insulin release in a PKA‐dependent manner,^7^, ^33^, ^34^, ^35^ we hypothesized that Phylloseptin‐PBu act as an agonist to GLP‐1 receptor and contributes to cAMP/PKA signalling activation. Following on from this, we used GLP‐1 receptor antagonist exendin‐(9‐39) to examine whether it can impair Phylloseptin‐PBu's action. As the results shown in Figure 6, the insulin secretion effect was significantly decreased in both exendin‐(9‐39) and exendin‐(9‐39) with Phylloseptin‐PBu co‐incubation groups; nonetheless, the latter group also showed a prominent higher insulin output. While further consistently activating K_ATP_ channels with diazoxide or blocking the calcium channels with verapamil showed an additive insulin secretion inhibition effect. In the meantime, the calcium deprivation patterns, again, exhibited significant lower insulin production, of note is that under such conditions, Phylloseptin‐PBu still stimulated higher insulin production. As a whole, these results confirmed that Phylloseptin‐PBu could interact with GLP‐1 receptor as a partial agonist to active PKA signalling for modulating Ca^2+^ channels, promoting insulin secretion. However, a Ca^2+^ and PKA signalling‐independent mechanism induced by Phylloseptin‐PBu was likely to play a role in this action.

### 3.7 Other mechanism examination for the induction of insulin release by Phylloseptin‐PBu

**Figure 7:**
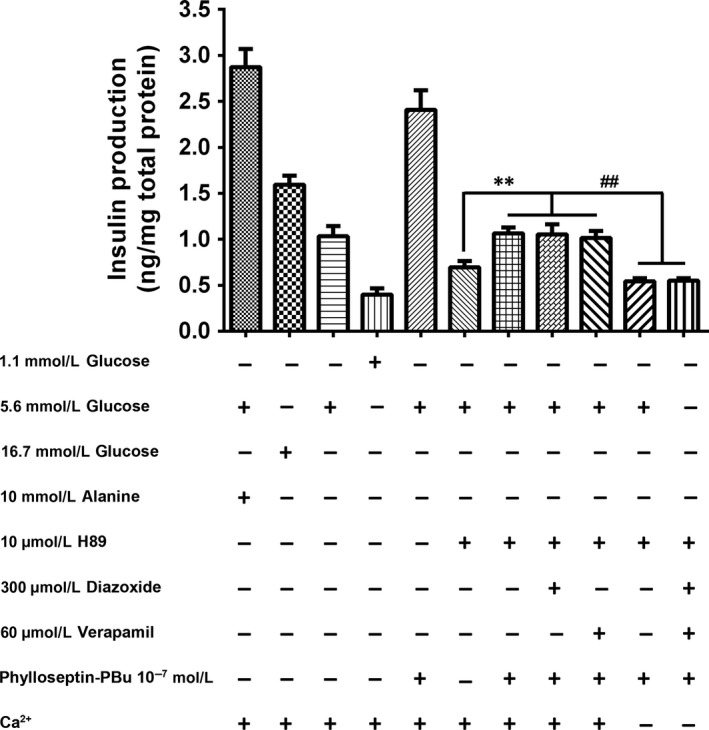
Acute effects (20 min stimulation) of 10^−7^ mol/L Phylloseptin‐PBu on the insulin release. BRIN‐BD11 cells were incubated with 1.1 mmol/L glucose, 5.6 mmol/L glucose, 16.7 mmol/L glucose, 10 mmol/L alanine, 10 μmol/L H89, 300 μmol/L diazoxide, 60 μmol/L verapamil and 10^−7^ mol/L Phylloseptin‐PBu in the Ca^2+^ present or absent KRB buffer. Data were analysed with unpaired Student's *t* test using GraphPad Prism 5 software. Values are the mean ± SEM for three independent experiments. ** or ^##^ *P* < .01 between indicated groups

In order to assess whether other signalling is existed in regulating Phylloseptin‐PBu's insulinotropic activity, we treated the cells using diazoxide or verapamil together with H89, and measured the insulin release. As the Figure 7 shown, compared with singular H89 treatment, Phylloseptin‐PBu still has moderately, but significantly higher insulin release activity even co‐treated with H89 and/or with diazoxide/verapamil, but this effect was completely abolished in the Ca^2+^‐free medium settings, which proposed a K_ATP_‐ and PKA‐independent Ca^2+^ influx mechanism.

## 4 DISCUSSION

In the postprandial state, elevated blood glucose is sensed by pancreatic beta‐cells which in response secrete insulin to maintain glucose homoeostasis. This process occurs under both physiological and pathological conditions, and thus, increasing insulin release has promising therapeutic potential.^5^

The mechanism correlated with acute insulin secretion in islets has been well‐studied, which is commonly summarized as two distinct pathways, one is related to the K_ATP_ channel closure, and membrane depolarization‐triggered intracellular calcium rise via Ca^2+^ channels on both cell membrane and endoplasmic reticulum, the other one is through Ca^2+^‐induced insulin secretory vesicle exocytosis.^31^ In this study, we investigated the K_ATP_‐channel, calcium channel, PKA signalling and GLP‐1 receptor in regulating Phylloseptin‐PBu‐induced insulinotropic effect in BRIN‐BD11 cells, we confirmed that this novel peptide stimulates acute insulin release at physiological concentration of 5.6 mmol/L glucose by deactivating K_ATP_ channels, leads to the opening of calcium channels and calcium influx, which subsequently drives the fusion of secretory vesicles to the plasma membrane for exocytosis of insulin. We also confirmed that Phylloseptin‐PBu partially stimulates GLP‐1 receptor, resulting in PKA activation for the increased insulin secretion. Notwithstanding, it must be noticed that, in Figure 5, Ca^2+^‐free medium remarkably decreased insulin output even under the condition of blocking calcium channel using verapamil, suggested a novel cytosol Ca^2+^ import mechanism, which is further supported by the independent results of Figure 7, and we suppose that ionotropic receptors (also known as ligand‐gated ion channels) might play the crucial role in it, which warrants further investigations. Moreover, based on Figure 5 and part Figure 6 results, the novel peptide still enhanced insulin release significantly compared with no Phylloseptin‐PBu treatment groups, which implied an intracellular Ca^2+^ store (endoplasmic reticulum)‐referred mechanism induced by Phylloseptin‐PBu. In addition, another interesting issue is that, in Figure 5A, we observed the additive augment insulin release effect of Phylloseptin‐PBu with IBMX, we presume that IBMX, as a phosphodiesterase inhibitor to raise intracellular cAMP,^36^ has distinct PKA activation mechanism with Phylloseptin‐PBu, which involves activating of GLP‐1 receptor, and therefore, the hyperactive PKA by both Phylloseptin‐PBu and IBMX contributed to the dramatically increased insulin secretion.

In consideration of the secondary structure of Phylloseptin‐PBu, which could also be crucial for its biological relevance, we conducted the CD analysis, the results showed that in 50% TFE aqueous solution, this novel peptide formed a well‐defined α‐helical structure, while in polar solvent, much less of (7%) α‐helicity was presented. This result was similar to glucose‐dependent insulinotropic polypeptide (GIP), GLP‐1, extendin‐4 and glucagon,^37^, ^38^, ^39^, ^40^ likewise, we assume that the full length of α‐helical conformation might be important for Phylloseptin‐PBu to exert the insulin induction activity. Nonetheless, further NMR or other structure‐activity relationship studies are needed for determination.

In addition, we also measured the insulinotropic activity of Phylloseptin‐PBu in 16.7 mmol/L glucose conditions to test whether it could further enhance insulin release, the results showed that no higher insulin release was observed (Figure S3). We speculate that the high glucose level might induce the cells to be stressful, and in such condition, the capacity of insulin release from cells could be limited by the high baseline insulin release.

Overall, in this project, we isolated and identified a novel peptide from *Phyllomedusa burmeisteri*, the insulinotropic effect of this novel peptide Phylloseptin‐PBu has been substantially studied, we confirmed the energy‐mediated K_ATP_ and Ca^2+^ channels, and the GLP‐1 receptor initiated PKA activation involvements in this effect, which has never been published from amphibian sources, our study continuously provide novel candidate and fundamental understanding for the treatment of T2DM in future.

## CONFLICT OF INTEREST

The authors declare that the research was conducted in the absence of any commercial or financial relationships that could be construed as a potential conflict of interest.

## AUTHOR CONTRIBUTION

Yuxin Wu, Tianbao Chen, Lei Wang and Mei Zhou conceived and designed the experiments. Qilin Long performed the experiments. Qilin Long and Yuxin Wu analysed the data. Tianbao Chen and Lei Wang contributed reagents/materials/analysis tools. Qilin Long and Yuxin Wu wrote the paper. Yuxin Wu and Tianbao Chen edited the manuscript.
