# Supplementary figures and images for: A novel peptide Phylloseptin‐PBu from Phyllomedusa burmeisteri possesses insulinotropic activity via potassium channel and GLP‐1 receptor signalling
Source: J Cell Mol Med. 2018 Mar 7;22(5):2804–14. doi: 10.1111/jcmm.13573 (PMC5908111; doi:10.1111/jcmm.13573)

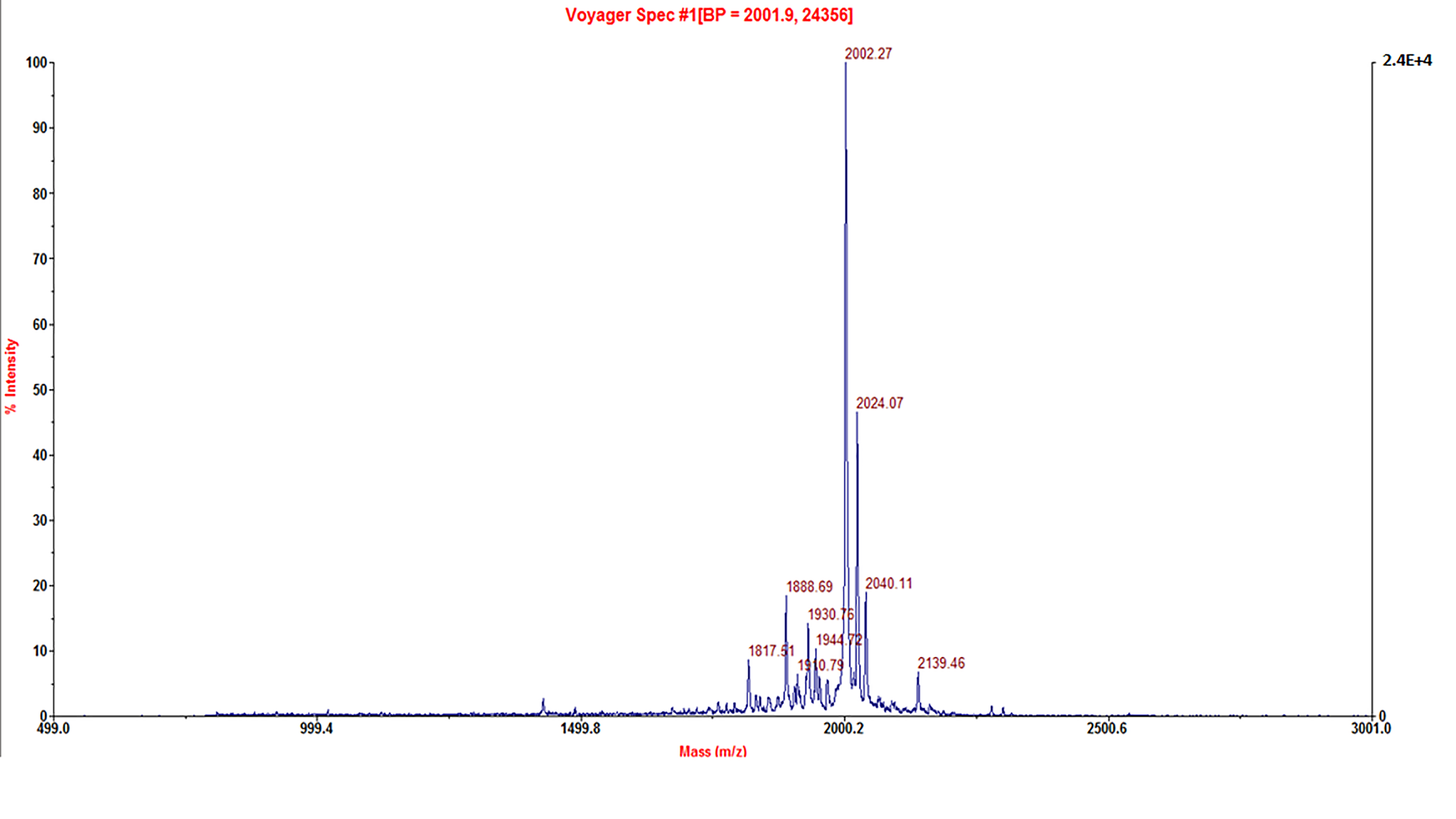

Supplement: Supplementary file 1 [file JCMM-22-2804-s001.tif]
